# Prevalence and determinants of test anxiety among medical students in Addis Ababa Ethiopia

**DOI:** 10.1186/s12909-019-1859-5

**Published:** 2019-11-14

**Authors:** Light Tsegay, Shegaye Shumet, Woynabeba Damene, Gebrselassie Gebreegziabhier, Getinet Ayano

**Affiliations:** 1 Department of Psychiatry, College of Health Sciences, Axum University, Axum, Ethiopia; 2 Research and training Department, Amanuel mental specialized hospital, Addis Ababa, Ethiopia; 3 School of Public Health, Curtin University, Perth, Australia

**Keywords:** Test anxiety, Prevalence, Medical students, Ethiopia

## Abstract

**Background:**

Worldwide, problematic test anxiety is a common health problem among medical students. The magnitude of problematic test anxiety ranges from 25 to 40% in undergraduate medical students and has a detrimental effect on academic achievement and success of students. However, data on the prevalence of test anxiety among medical students is limited. Thus, the study aimed to assess the prevalence and associated factors of test anxiety among medical students.

**Methods:**

In this cross-sectional study, a stratified random sampling technique was used to select the participants. The level of test anxiety was determined by the Westside Test Anxiety Inventory (WTAI). We utilized logistic regression to explore the association between test anxiety and the potential sociodemographic/student-related characteristics among medical students.

**Results:**

The study included 423 medical students. Our study resulted the prevalence of problematic test anxiety among medical students to be 52.30% (95% CI 47.40–57.30). The prevalence of test anxiety was remarkably higher in women (79.75%) than in men (33.62%) students. Female sex [AOR = 3.25, 95% CI: (1.54, 6.89)], having low grade [AOR = 0.11,95% CI: (0.044,0.288)], being first year [AOR = 10.55,95% CI: (1.4,76.7)], excessive course load [AOR = 6.128,95% CI: (2.675,14.039)], and taking oral examination [AOR = 2.89,95% CI: (1.42,5.84)] were determined as some of the predicting factors of test anxiety among medical students. Additionally, lack of systemic study plan [AOR = 2.4, 95% CI: (1.25, 4.59)], poor social support [AOR = 3.6, 95% CI: (1.56, 8.29)], moderate social support [AOR = 3.39, 95% CI: (1.56, 7.4)], psychologically distressed [AOR = 2.68, 95% CI: (1.37, 5.27)] independently predicts test anxiety among medical students.

**Conclusion:**

Findings suggest that a substantial percentage of medical students had problematic test anxiety in Ethiopia (52.30%). This study also showed a significant association between test anxiety and female sex, having poor grade point average, being the first year, excessive course load, oral examination, lack of study plan, poor social support, moderate social support, and having psychological distress. Problematic test anxiety, which is found to be common among medical students, deserves more attention.

## Background

Test anxiety is referred to as the set of psychological and behavioral responses that accompany concern about likely negative consequences or failure of an exam or similar evaluation situations [1]. Test anxiety is a situation-specific trait that refers to the anxiety states and worries conditions that are happened during examinations. This type of anxiety appeared abruptly or gradually. Sometimes it is persistent, and sometimes or ended within a few hours [2–4].

Literature shows that most students experience test anxiety during exam but when the anxiety interferes with the student’s capacity to perform in exam adequately and express their knowledge on examinations, it becomes a problem [5]. In fact, test anxiety is a great obstacle in the way of many individuals to reach their real academic destination [6].

Test anxiety can be diagnosed using the Diagnostic and Statistical Manual-IV, under the classification of social phobia and has emotional and cognitive components [7]. Cognitive test anxiety is the worry part of cognitive reaction or internal dialogue in the lead up to, during and after evaluative situations, which is correlated with academic performance. Individuals who have high levels of cognitive test anxiety often report feelings of comparing self-performance to peers, worries over the consequences of failure, low levels of confidence in their own abilities as well as too much worry over evaluation. Additionally, the emotional component (sometimes called tension) refers the sharp physiological symptoms stemming from arousal of the autonomic nervous system and related affective responses that students have during the test, which is physiological manifestations include increased galvanic skin response and heart rate, muscle tension dizziness, nausea, or feelings of panic [3, 8].

According to educational psychologists and experts in education, an average level of anxiety is useful as an effective motivational factor can enhance one’s performance for more effort. However, excessive anxiety can result in different effects like disturbance of mental processes [4].

Evidence also suggests that medical school programs are characterized by challenging classes and high credit loads in each academic term, which in turn is associated with higher rates of test anxiety, resulting poor academic performance and higher school dropout rates [1]. Epidemiologic studies shows that 25 to 40% of undergraduate students experience test anxiety [9, 10].

Several studies revealed that the major factors associated with test anxiety were female sex, inability to manage time, and lacking study skills, low previous grade (GPA), psychological distress, poor self-esteem, and poor social support [3, 11–16]. Excessive course load, lack of revision time before the exam [5, 16]. In addition, there is variation across faculty, having low academic competence and test competencies were the significant predictors of test anxiety [5, 16].

To the best of our knowledge, there are no previous studies that explored the prevalence and associated factors of test anxiety in undergraduate medical students in Ethiopia. Therefore, this is the first study to assess the prevalence of test anxiety and its determinants in undergraduate medical students in Ethiopia. Additionally, the study is important for a better understanding of the level of test anxiety and its predictors, which enables the responsible stakeholders to intervene, based on the findings.

## Methods

### Study design and population

This was an institutional-based cross-sectional study conducted from May to May 2018 at Addis Ababa University (AAU). Of 1650 medical students, 390 of them were randomly selected and completed the required questionnaires. All undergraduate medical students were eligible to participate in the current study.

### Sample size determination and sampling techniques

The sample size (n) to estimate the prevalence of test anxiety among medical students was determined by a single population proportion formula. 95% confidence level was considered to estimate the sample size and since there are no previous similar studies conducted in Ethiopia, we assumed the prevalence of test anxiety 50% and a precision of 5% between the sample and the parameter was taken. α =0.05 (95%) =1.96.

We employed the following sample size calculation formula for a single proportion:

$$ n=\frac{{\left(Z\frac{\alpha }{2}\right)}^2p\ \left(1-\mathrm{p}\right)}{d^2} $$

Where;*n* = sample size

$$ Z\frac{\propto }{2}=\mathrm{significance}\ \mathrm{level}\ \mathrm{at}\propto =0.05 $$

The *p* = expected proportion of test anxiety among undergraduate medical students supports to be 50% since no related study is found in Ethiopia.

d = margin of error = 0.05.

Therefore,

$$ n=\frac{(1.96)^20.5\left(1-0.5\right)}{0.05^2}=384 $$

Assuming the non-respondent rate 10% of, the final sample size was 384 + 39 = 423.

Additionally, we calculated sample size based on the potential associated factors using STATA 14.2 for sample size calculations, assuming the proportion of test anxiety 50% (no similar study in the area) and taking the odds ratio of 2. In addition, we assumed a significant level of 5 and 80% power. The total sample size was 296 (148 per groups). Adding 10% non-response rate the sample was 320 for each associated factors. However, we used the larger sample size (calculated based on proportion, *n* = 423) for this study.

**Fig. 1:**
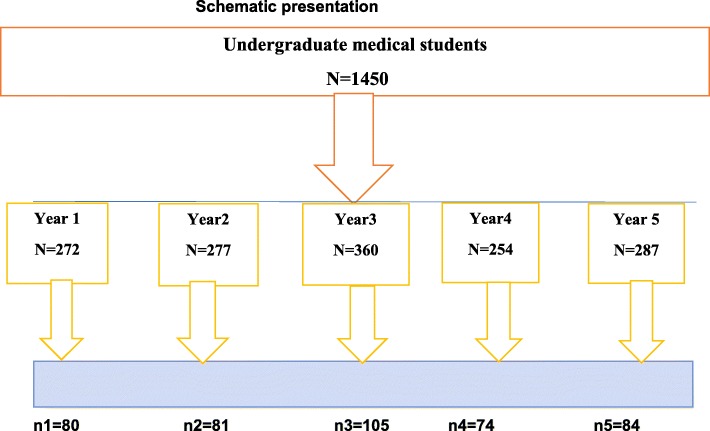
Schematic presentation of sampling technique (*n* = 390)

Concerning the sampling technique, in this study, a total of 390 participants, were randomly selected by using a stratified random sampling technique. As suggested, we used a proportionate stratified random sampling to allocated sample for students in each year (year 1 to 5) and we employed a simple random sampling method to select each participant. (See Fig. 1**).**

### Measures

#### Measures for the dependent variable (test anxiety)

Data was collected by a self-administrated questionnaire. Westside Test Anxiety inventory developed by Richard Driscoll (WTAI), is a self-reported questionnaire of 10 statements to which respondents are asked to report how often they experience anxiety symptoms before, during, and after taking tests. Each statement response is scored with a 5-point Likert scale [1–5] yielding a total test anxiety score ranging from 10 to 50 points. Participant were attributed to five different levels of test anxiety according to WTAI score: score of 1.0–1.9 comfortably low-test anxiety, a score between 2.0—2.5 Normal or average test anxiety, 2.5—2.9 High normal test anxiety, 3.0—3.4 Moderately high,3.5—3.9 High test anxiety,4.0—5.0 Extremely high anxiety and the cut point for problematic test anxiety is ≥30. The Cronbach alpha was alpha of .78, split-half reliability of .77 in a Nigerian sample [17, 18].

Therefore, In this study, those students who scored below 30 were considered as having no test anxiety and those score 30 and above were classified as having test anxiety [18]. In our sample, the internal consistency of WTAI was very high (Cronbach’s alpha =0.94).

### Measures for the predictor variables

#### Study skills

Study skills referred to as **t**he ability to effectively use the specific skills (learning and planning study, library use, note-taking, course participation, preparation for exams, motivation, preparation for courses, effective reading, writing, health and nutrition, and listening skills). Time Management (TM) includes six items inventory was used in this study to measure the time management of students it has 6 questions and 5 points Likert scale<20 suggest not effective time management skill [19].

To assess the study skills we used approaches and a study skills inventory for students (ASSIST) [19]. The inventory consists of 51 items 5-point Liker scale. It measures the scores on six subscales regarding study habits and skills. These subscales are Textbook Reading, Note-Taking, Memory, Test Preparation, Concentration and Time Management. The domain of Time Management (TM) includes six items of the inventory. The convergent validity of the tool is high and consistency reliability is 0.96 which is.

#### Self-esteem

Rosenberg Self-esteem scale (RSES) was used to measure levels of self-esteem among the study participants [20]. Students were scored based on a 4-point Liker scale, scoring of the scale “Strongly Disagree” 1 point, “Disagree”2 points, “Agree” 3 points, and “Strongly Agree” 4 points. The sums of the scores for all ten items and keep them on a continuous scale. Higher scores indicate higher self-esteem. The scale ranges from 0 to 30. Scores between 15 and 25 are within normal range; scores below 15 suggest low self-esteem. Internal Validity is 0.78–0.84 And reliability is 0.94 which is high [20].

#### Psychological distress

Psychological distress was measured using the Kessler Psychological Distress Scale (K-10). It is a simple measure of psychological distress, the K10 scale involves 10 questions about emotional states each with a five-level response scale. According to the scale, a score 10–19 Likely to be well, 20–24 Likely to have a mild disorder, 25–29 Likely to have a moderate disorder, 30–50 Likely to have severed distress. The scale has a sensitivity of 70% and specificity 67% [21].

#### Social support

We also measured social support of the students and individual who were scored 3–8 poor, 9–11 moderate and 12–14 strong social support on Oslo 3-item social support scale (OSSS-3) [22]. Evidence suggests that the OSSS-3 is a very brief and economic scale which has satisfactory internal consistency with an α = .640 [23]. Although OSSS-3 is not validated in Ethiopia, it is extensively used in previous studies to assess social support in different population groups [24–26].

### The sociodemographic and other variables

We collected self-reported data on sociodemographic and other characteristics of the participants using self-reported and structured questioners.

### Data collection procedure

In this study to maintain the quality of the data high, before they engage in the actual data collection activities the data collectors received adequate training on data collection procedure and protocol. Data was collected by using an English version of self-administrated questionnaire.it had components which assess a different aspect of the participant. The 1st part assessed a socio-demographic characteristic of participants. The 2nd part assessed the level of test anxiety by using the WTAI scale. The 3rd part was on psychosocial factors (psychological distress, self-esteem, and social support) which affect the level of test anxiety and measured by K-10 scale, RSES and oslo3-social support scale respectively. The 4rth part was on questions assessing behavioral factors using SSI scale to assess time management. The fifth part assessed the academic-related factors that affect test anxiety using yes or no questions. This questionnaire disseminated to 423 eligible medical students.

### Data quality assurance

The questionnaire was designed and modified appropriately. The self-administrated English version was disseminated. The training was given for data collectors and supervisor. Pre-test conducted 2 days before the actual data collection on saint Paulo’s medical school and the Pre-test was conducted among 22 samples (5%) of saint Paulo’s medical students; the result was not included in the main survey.

Based on the finding from the pre-test, the questioner revised and helped to estimate the time needed for data collection. The data collectors were supervised daily and assist the students to fill the questionnaire and checked daily by the supervisors and principal investigator. The solution to problems during data collection was given immediately by discussing with the supervisors and the data collectors.

### Data processing and analysis

First, the data were checked for completeness and consistency and then coded and entered in the computer using EPI DATA software for cleaning, storing and recording and then imported to SPSS version 25 for analysis. Descriptive statistic was used to explain the study participants in relation to study variables. A *p*-value of less than 0.05 was considered statistically significant. Bivariate and multivariate logistic regression analysis was conducted to identify associated factors of test anxiety among medical students and the strength of the association was presented by odds ratio with a 95% confidence interval.

### Ethical consideration

This cross-sectional study was reviewed and approved by the Institutional Review Board (IRB) of the University of Gondar and Amanuel Mental Specialized Hospital. Informed consent from each participant was obtained after clearly explaining the objectives as well as the significance of the study for each study participant. We advised the study participants about the right to participate as well as refuse or discontinue participation at any time they want and the chance to ask anything about the study. The participants were also advised that all data collected would remain confidential.

## Result

### Socio-demographic characteristics of respondents

Of the total 423 participants reached by random sampling, 390 participants were involved in this study with a response rate of 92.2%. The population was (40.5%) female and (59.5%) male and with a mean of 21.8 years with SD (±1.72) ranging 18 to 26 years.

More than half of the participants 245 (62.8%) were Orthodox by religion and most of the respondents were single 385(98.7%) and regarding education level of the respondent’s majority of the participant were 3rd year 92(23.6%) followed by 2nd year 80(20.5%). **(**Table 1**).**

**Table 1:** Socio demographic characteristics of medical students at AAU, Tikur Anbessa specialized Hospital, Addis Ababa, Ethiopia, 2018 (*n* = 390)

Variable	Category	Frequency	Percentage
Age	Mean	Standard deviation	
	21.8	±1.72	
Sex	Female	158	40.5
	Male	232	59.5
Religion	Orthodox	245	62.8
	Muslim	38	9.7
	Protestant	89	22.8
	Others*	18	4.6
Marital status	Single	385	98.7
	Married	5	1.3
Educational status	First year	74	19.0
	Second year	80	20.5
	Third year	92	23.6
	Fourth year	73	18.7
	Fifth year	71	18.2

*Catholic, Jova witness and seventh Adventist

### Academic characteristics

Out of 390 students, 192(49.2%) of them reported maternal educational status as tertiary and above, (75.6%) of them reported history of anxiety that increased during oral exam and (24.4%) reported history of anxiety that increased during a written exam, (43.1%) faced excessive course load, and (73.8%) of them lack enough time to revise before exam. (Table 2).

**Table 2:** Academic characteristics of undergraduate medical students at Tikur Anbessa Specialized Hospital Addis Ababa, Ethiopia, 2018 (*n* = 390)

Variable	Category	Frequency	Percentage
Grade Point average	Mean	Standard deviation	
	3.38	***±***0.48	
Maternal educational status	Less than primary	40	10.3
	primary	73	18.7
	secondary	85	21.8
	tertiary and above	192	49.2
Examination type	oral exam	295	75.6
	written exam	95	24.4
Excessive course load	Yes	168	43.1
	No	222	56.9
Enough revision time	No	73.8	73.8
	Yes	26.2	26.2
Study plan	No	297	76.2
	Yes	93	23.8

### Psychosocial characteristics of participants

In this study, 186 (47.7%) of the participant had psychologically distressed. Regarding social support 138 (35.38%) and 166 (42.57%) had poor and strong social support respectively. In this study, 337 (86.41%) of the participant had high self-esteem. (Table 3).

**Table 3:** Psychosocial characteristics of undergraduate medical students at Tikur Anbessa Specialized Hospital Addis Ababa, Ethiopia, 2018 (*n* = 390)

Variable	Category	Frequency	Percentage
Psychological distress	Yes	186	47.7
	No	204	52.3
Social support	poor	138	35.38
	moderate	86	22.05
	strong	166	42.57
Self-esteem	low	8	2.05
	average	45	11.54
	high	337	86.41

### Behavioral characteristics of the participant

The results of this study found that about 10 (2.6%) of the participant had attempted suicide in their lifetime. Regarding time management, 267(68.5%) of students had poor time management and 24.1% of the participant used the internet more than 5 h per day and 22.8% used more 30 min to 1 hour per day**.** (Table 4).

**Table 4:** Behavioral and clinical characteristics of undergraduate medical students at Tikur Anbessa Specialized Hospital Addis Ababa, Ethiopia, 2018

Variable	Category	Frequency	Percentage
Suicidal attempt	Yes	10	2.6
	No	380	97.4
Time management	Poor	267	68.5
	Good	123	31.5
Internet use habit	more than 5 h	94	24.1
	3 h–4 h	121	31.0
	1.30 min-2 h	86	22.1
	3omin-1 h	89	22.8

### Prevalence of test anxiety among medical students

In this study, more than half (52.30% (95%CI 47.40–57.30)) of medical students had problematic test anxiety. The prevalence of test anxiety in females (79.75%) was remarkably higher than in males (33.62%).

### Factors associated with the level of test anxiety among medical students

In this study, female sex, having a poor average grade, being the first year, excessive course load, oral examination, lack of study plan, poor social support, moderate social support, and psychological distress were significantly associated with test anxiety in our final multivariable logistic regression. The final model has the Cox and Snell R-square of 0.523 and 0.79 Neglekerke R-square. The Hosmer and Lemeshow test was not statistically significant (*P* = 0.289).

The odds of having positive test anxiety for a female is 3.25-fold higher than male [AOR = 3.25, 95% CI: (1.54, 6.89)]. Regarding grade of students, when the average grade point increase in a unit, the odds of developing severe test anxiety decreased by 88.8% [AOR = 0.11,95% (0.044,0.288)].

We also found that the risk of developing positive test anxiety for first-year students is 10 times higher than the fifth-year students [AOR = 10.55, 95% (1.4, 76.7)]. Having excessive course load was 6 times higher risk to develop positive test anxiety [AOR = 6.128,95% (2.675,14.039)].

Furthermore, the odds of having positive test anxiety for an oral examination is 2 times higher than that of written examination. [AOR = 2.89,95(1.42,5.84)]. The odds of having severe test anxiety for students who did not have a systemic study plan is 2.4 higher than those who had a study plan [AOR = 2.4,95% (1.25,4.59)]. The odds of having severe test anxiety for those who have poor social support is 3.6 higher than those who have strong social support [AOR = 3.6,95% (1.56,8.29)].

Additionally, the odds of having positive test anxiety for those who have moderate social support is 3.39 higher than those who have strong social support [AOR = 3.39,95% (1.56,7.4)]. The odds of having severe test anxiety for those psychologically distressed students is 2.68 times higher than those not distressed [AOR = 2.68,95% (1.37,5.27)] (Table 5).

**Table 5:** Factors associated with level of test anxiety among undergraduate medical students at Tikur Anbessa Specialized Hospital (Bivariate and multivariate analysis) (*n* = 390), Addis Ababa, Ethiopia, 2018

Variable	Test anxiety		COR (95%CI)	AOR (95%CI)
	No	Yes		
Age	mean	standard deviation		
	21.18	1.719	0.83 (0.73,0.93)	1.084 (0.71,1.64)
Sex				
Female	32	126	7.77 (4.8, 12.4)	3.25 (1.54, 6.89)
Male	154	78	reference	
Grade point average	mean	standard deviation		
	3.38	±0.48	0.03 (0.02,0.05)	0.11 (0.044,0.288)
Educational level				
1st year	20	54	3.69 (1.83,7.4)	10.55 (1.4,76.7
2nd year	37	43	1.58 (0.83,3.02)	1.81 (0.339,9.7)
3rd year	49	43	1.19 (0.64,2.23)	1.68 (0.49,5.76)
4th year	39	34	1.19 (0.61,2.3)	0.5 (0.168,1.49)
5th year	41	30		
Maternal educational status				
< Primary	16	24	1.5 (0.75,2.99)	1.89 (0.628,5.73)
Primary	35	38	1.08 (0.63,1.86)	1.07 (0.42,2.67)
Secondary	39	46	1.17 (0.7,1.96)	1.63 (0.69,3.86)
Tertiary	96	96	reference	
Exam type				
Oral	108	187	7.94 (4.46, 14.1)	2.89, (1.42, 5.84)
Written	78	17	reference	
Course load				
Yes	42	126	5,53 (3.55,8.64)	6.128,(2.675,14.039)
No	144	78	reference	
Study plan				
No	110	187	7.6 (4.27,13.5)	2.4 (1.25,4.59)
Yes	76	17	reference	
Social support				
Poor	20	118	19.2 (10.6,34.8)	3.6 (1.56,8.29)
Moderate	39	47	3.92 (2.25,6.84)	3.39 (1.56,7.4)
Strong	127	39	reference	
Internet use				
30 min-1 h	45	44	reference	
1.30 min-2 h	38	48	1.29 (0.71,2.34)	0.98 (0.37,2.59)
3-4 h	53	68	1.31 (0.75,2.27)	1.85 (0.702,4.88)
> 5 h	50	44	0.9 (0.5,1.6)	1.72 (0.62,2.5)
Time management				
Poor	101	166	3.67 (2.33,5.79) q	1.25 (0.62,2.5)
Good	85	38	reference	
Psychological distress				
Distressed	49	137	5.71 (3.68,8.85)	2.68 (1.37,5.27)
Not distressed	137	67	reference	

## Discussion

### Main findings

To the best of our knowledge, this is the first study that assessed the prevalence of problematic test anxiety and determinants among medical students in Ethiopia. Findings from the present study demonstrated that more than half of the medical students had problematic test anxiety (52.30%). This prevalence is consistent with the reported magnitude of test anxiety from Saudi Arabia (53%) [27], the USA (55%) [28], and Turkey (48%) [29].

However, the result of the current study was higher than the study conducted at Iran ((43.4%) [30], Malaysia (32.5%) [2], and India (32.3%) [31]. This discrepancy might be due to the sampling size difference, the methodological differences including the instrument used to measure test anxiety and differences in the study in the characteristics of the population in each country.

On the contrary, the result of this study is lower than the study conducted in Saudi Arabia and Kenya with the prevalence of (65%) [32] 68.1%) [33] respectively**.** This difference may be due to the variations in several factors that have an impact on anxiety, such as different course contents, educational environment, test conditions, types of test questions and other factors.

In this study, the prevalence of test anxiety was found to be more than two-fold higher in female students as compared to male students. This result is supported by a study carried out in Sudan and Pakistan [34, 35]. The possible reason for this strong association can be due to the reason that all night studying before exams is significantly higher among female students, as compared to male, this can create fatigue and overall exertion among students which may lead to a lower performance in examinations or due to females over-report their problems than males [36].

On the other hand, the present finding does not agree with the results the study conducted in Iran [10] where no significant association between test anxiety and gender was reported and a study in India [37] where test anxiety is higher in males compared to female students. The discrepancy might be due to variation in the study area and the characteristics and type of the study participants because in the above study the study participants were high school students and the study involved greater number of male than female participants. Biological (including defense mechanisms) [38–41] and environmental factors such as parental pressure [42], previous poor performance, fear of failure and procrastination, expectations, and preparations [37, 43] as well as the nature and the characteristics of the test [43, 44] are the possible explanations for the higher magnitude of test anxiety among the medical students. Additionally, as described by Yerkes-Dodson law, the higher level of test anxiety could be due to an excessive level of arousal which potentially leaving them nervous and unable to concentrate on the test [45].

Regarding the associated factors, this study showed that the score of the students was found to be a significant predictor for test anxiety among medical students. Higher-grade scores was associated with increased odds of developing test anxiety among medical students. This result is supported by studies conducted in Saudi Arabia, Sri Lanka and Nigeria [14, 46, 47] where higher-grade scores were associated with greater risks of test anxiety. On the other hand, a study conducted in Iran showed no significant association between GPA and test anxiety [48]. The discrepancy could be due to differences between universities educational environment, teaching and evaluation methods, and systems of rewards and punishments resulting from test results play a role in this regard.

Furthermore, the year of students was also found to be a significant predictor of test anxiety among medical students. The odds of developing test anxiety was 7.0 times higher among first-year medical students as compared to fifth-year students. This might be due to substantial stress at the beginning of the course with more concern and uncertainty about their academic performance at the early stages of the study. The finding of this study is in agreement with studies done in India [31]. Conversely, a study conducted in Saudi Arabia among female medical students showed a greeter odds of test anxiety for higher year students as compared with first-year students [11] test. The variation could be due to the study was conducted on female medical students only or due to curriculum difference.

In the present study, test anxiety for an oral examination was 5.5 times higher as compared with a written examination. This finding is in line with the results from studies conducted in Germany and India [49, 50]. The possible reason might be due to oral exams require additional skills such as language, social interaction, and communication skills as compared to written exams, which possibly increase the anxiety.

In the current study, the level of test anxiety for students who faced extensive course, the load is four times higher than those who did not face excessive course load. This is in line with studies conducted in Pakistan and Saudi Arabia [32, 51] where extensive course load contributes 90.5 and 23.4% for exam anxiety, it can be due to the reason that since the medical course is so vast and examination process is so lengthy, students’ perception of course load and their ability to manage time with their course work is associated with exam anxiety [32].

Moreover, in this study, test anxiety for students who did not have a systemic study plan was two times higher than those who had a study plan. This results is in line with the reported result from a study conducted in Pakistan [52]. The possible reason can be due to Ineffective study habits leads to poor preparation and students encode and store the material inadequately, as a result, they are unable to recall poorly learned material during the examination.

The odds of having high-test anxiety for those who had poor social support is three times higher than those who had strong social support. This finding is in agreement with the findings from studies conducted in Turkey and Iran [13, 53]. Having someone around when needed and participating in social activities decrease students’ emotional tension and release energy, which in turn decrease anxiety.

Finally, the odds of developing test anxiety was three times higher among students who had psychological distress as compared to those students who had no psychological distress. This result is in line with the reported results from studies conducted in Saudi Arabia [11, 54] where a significant and positive association between psychological distress and test anxiety was identified.

## Conclusion

This study showed that the average prevalence of test anxiety was high among undergraduate medical students (52.30%). Female sex, being the first year, lower grade point average, oral exam, excessive course load, lack of systemic study plan, lower social support and psychological distress were significantly associated with the level of test anxiety. Early screening and interventions of test anxiety among medical students were warranted.

## Data Availability

The datasets used and analyzed during the current study are not publically available due to ethical restriction and personal data protections but are available from the corresponding author on reasonable request.

## Abbreviations

ASSIST: Approaches and Study Skills Inventory for Students
IRB: Institutional Review Board
K-10: Kessler Psychological Distress Scale
RSES: Rosenberg Self-esteem scale
SPSS: Statistical Software for Social Science
TM: Time Management
WTAI: Westside Test Anxiety inventory
